# Non-Coding RNAs Implicated in the Tumor Microenvironment of Colorectal Cancer: Roles, Mechanisms and Clinical Study

**DOI:** 10.3389/fonc.2022.888276

**Published:** 2022-04-28

**Authors:** Zhaoxu Wu, Qiang Ju

**Affiliations:** Department of Blood Transfusion, The Affiliated Hospital of Qingdao University, Qingdao, China

**Keywords:** colorectal cancer, tumor microenvironment, non-coding RNAs, mechanism, clinical study

## Abstract

Colorectal cancer (CRC) is one of the most common malignant tumors. The morbidity and mortality rates have been increasing all over the world. It is critical to elucidate the mechanism of CRC occurrence and development. However, tumor microenvironment (TME) includes immune cells, fibroblasts, endothelial cells, cytokines, chemokines and other components that affect the progression of CRC and patients’ prognosis. Non-coding RNAs (ncRNAs) including microRNAs (miRNAs), long non-coding RNAs (lncRNAs), circular RNAs (circRNAs) without protein-coding ability have been shown to engage in tumor microenvironment-mediated angiogenesis and metastasis. Therefore, clarifying the mechanism of ncRNAs regulating the microenvironment is very important to develop the therapeutic target of CRC and improve the survival time of patients. This review focuses on the role and mechanism of ncRNAs in the CRC microenvironment and puts forward possible clinical treatment strategies.

## 1 Introduction

Colorectal cancer (CRC) is the most common malignant tumor, which seriously threatens human health (1). The incidence rate of CRC occupies the third position in all cancers worldwide (2). In addition, CRC is the second leading cause of death in malignant tumors due to the poor prognosis and high postoperative metastasis rate (3). Therefore, it is significant to clarify the mechanism of CRC progression and find treatment targets.

Tumor microenvironment (TME) includes immune cells, fibroblasts, endothelial cells, and other components that affect the occurrence and development of CRC (4). Cancer cells can functionally shape their microenvironment by secreting various cytokines, chemokines, and other factors (5). Existing evidence confirms that there are complex and continuous interactions between tumor cells and their microenvironment (6). Targeting TME can regulate multiple stages and processes in tumorigenesis and development (7). In recent years, many studies have shown that some ncRNAs are involved in the progression of CRC as an oncogene or tumor suppressor (8–10). Various types of ncRNAs are abnormally regulated in many cancer types (10, 11). They regulate proliferation, differentiation, apoptosis, necrosis, and autophagy of tumor cells by affecting the components of the TME (12). Therefore, the regulatory relationship between ncRNAs and TME has attracted more and more attention.

In this review, we describe the roles of abnormal expression of ncRNAs in the TME to promote CRC progression. Besides, we discuss the promising clinical treatment of ncRNAs in TME of CRC.

## 2 TME

TME is the internal environment in which tumor cells produce and live (5). It is mainly composed of tumor cells and their surrounding immune and inflammatory cells, tumor related fibroblasts, nearby interstitial tissues, capillaries, as well as various cytokines and chemokines (12, 13). The dynamic changes of the above components and other factors related to TME, such as hypoxia, chronic inflammation and immunosuppression promote the tumor progression and patients’ prognosis (14). The development of the tumorigenic microenvironment in CRC is driven by the genetic instability of its cancer cells and epigenetic factors in response to exogenous and endogenous stress stimuli (15–18). When intestinal inflammation occurs, various cells such as neutrophils, macrophages and fibroblasts are recruited (19). Fibroblasts and inflammatory cells will infiltrate into inflamed tissues and play a role in TME. These recruited inflammatory cells interact with CRC cells by producing various cytokines and chemokines to promote tumor growth and progression (20, 21). Actually, TME varies depending on the location and type of tumors. Its heterogeneity and dynamic changes lead to the therapeutic drug resistance of tumors (22). Therefore, an in-depth understanding of TME may provide important clues for finding new treatment options and enhancing treatment effects (23, 24).

## 3 Roles and Mechanism of ncRNAs in TME

**Figure 1:**
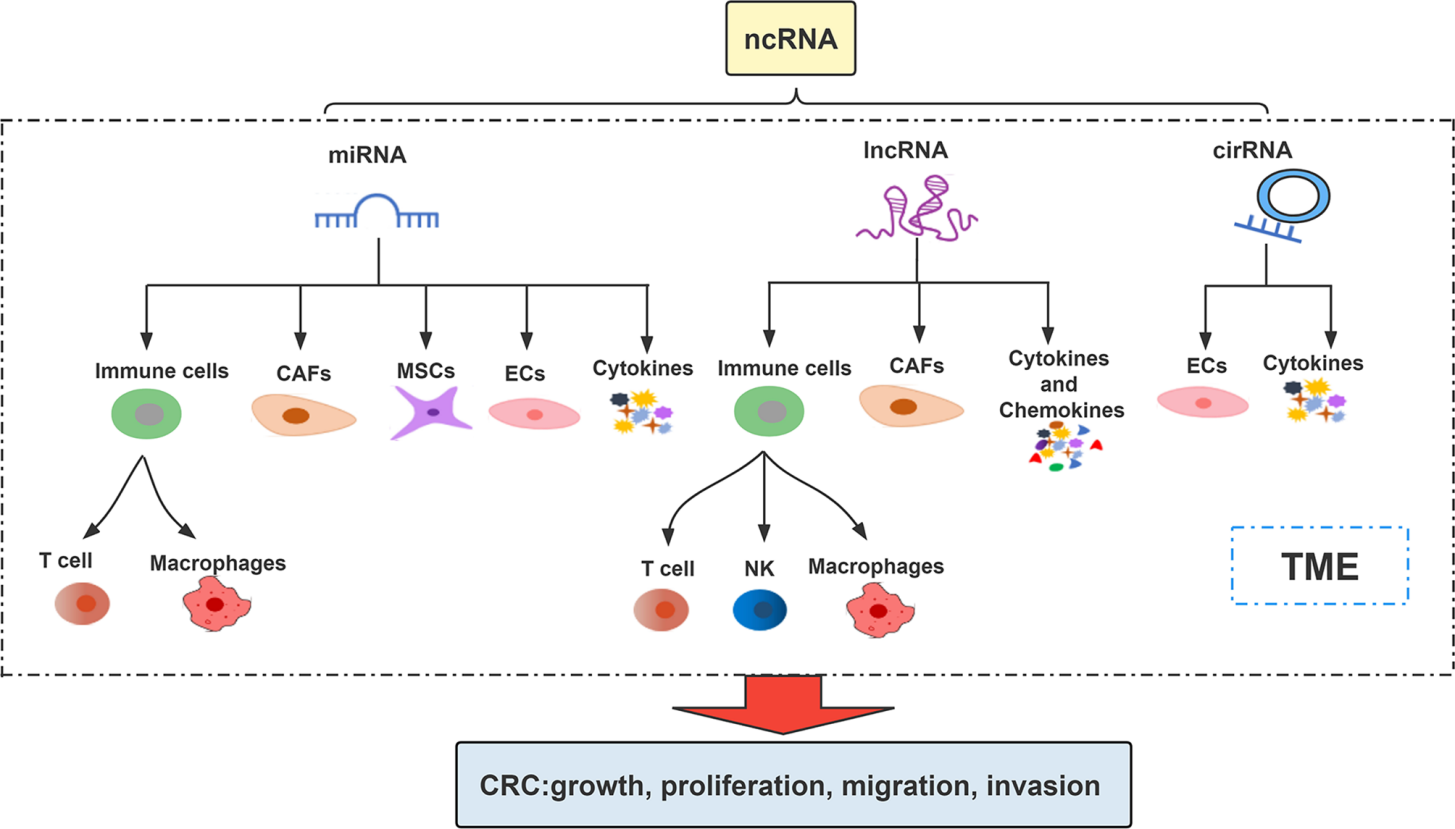
Important roles of ncRNAs in the TME.

ncRNAs are involved in the regulation of multiple biological processes by regulating gene expression in multi-level and multi-channel (25). They affect and participate in the regulation of CRC progression by affecting different cells or intercellular matrix, microvessels and various factors in TME (26, 27). The interaction among ncRNA, TME components and cancer cells increases the cancer progression (28) (**Figure 1**). Association between them makes ncRNAs (including miRNAs, lncRNAs, circRNAs) mediated TME as current potential treatment strategies for cancer treatment (29, 30).

### 3.1 miRNAs in TME

**Figure 2:**
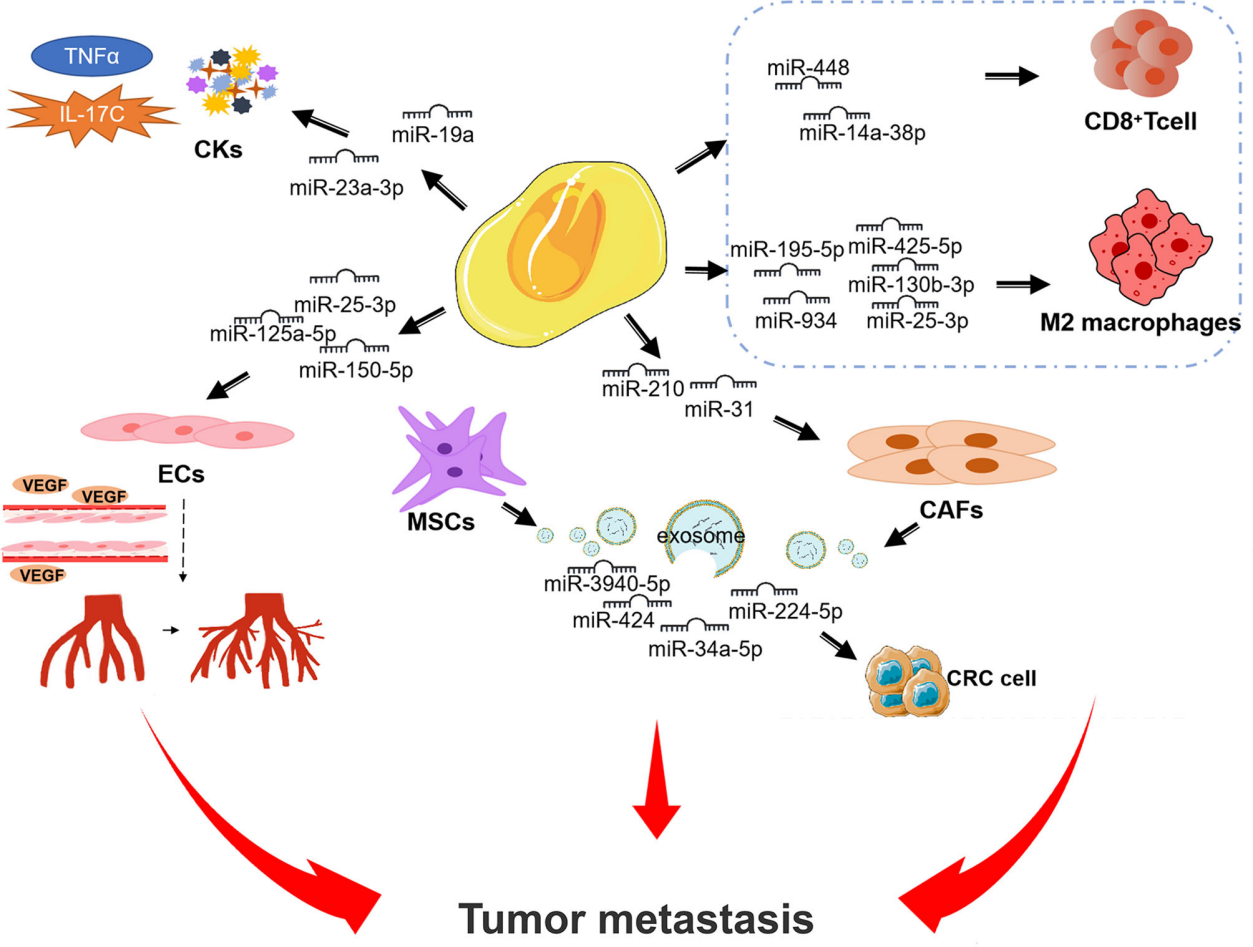
miRNAs affect the progression of CRC mediated by different components in TME.

miRNA is a type of small ncRNA that can bind to 3’-untranslated regions (3’-UTRs) or amino acid coding sequences to regulate mRNA expression (31). A large number of studies have shown that miRNA imbalance can play a role in many biological processes such as tumor proliferation, angiogenesis, metastasis, immune response and drug resistance through the interaction among malignant cells, non-malignant stromal cells and non-cellular components in TME (32, 33). It is worth noting that studies have confirmed that some miRNAs are abnormally expressed in CRC and are involved in the regulation of CRC immune escape (34, 35). Next, we summarize some miRNAs that influence the development of CRC through the different components of TME (**Figure 2**).

#### 3.1.1 Immune Cells

Immune cells are important parts of the TME (36). Inflammation and infiltration of immune cells into tumor tissues have been proven to support tumor growth, invasion and metastasis. Tumor progression can promote TME remodeling through the communication between tumor and immune cells (37). Different immune cells have the property of inhibiting tumors or promoting tumors, which depends on the stimulation of the cell and tissue environment (20, 37–39). In particular, the functional inactivation of tumor-reactive T cells play an important role (40–42). As an immunomodulatory enzyme, IDO1 can catalyze the degradation of tryptophan (Trp) to canine purine (Kyn) to induce apoptosis/dysfunction of effector T cells and generate immunosuppressive regulatory T cells (43–45). Lou et al. found that miR-448, which has a tumor-suppressive effect that can inhibiting the apoptosis of CD8^+^ T cells by inhibiting IDO1 expression in TME of CRC. That enables to exert cytotoxic T lymphocyte effector function and inducing tumor cells apoptosis (45). Conversely, miR-148a-3p affects CRC progression by inhibiting CANX/MHC-I axis and significantly attenuating CD8^+^ T cell-mediated immune attack *in vitro* and *in vivo* (46).

Macrophages are generally considered as the most common leukocytes in TME, which can be divided into M1-like macrophages (M1 macrophages) and M2-like macrophages (M2 macrophages) (47). Macrophages in TME are usually called tumor-associated macrophages (TAMs) (48) and most of them are M2-like (49). Programmed tumor cells can secrete mediators to activate tumor-associated macrophages (50). They respond to various factors produced by tumor cells in TME. These factors can induce epithelial mesenchymal transition (EMT) and play an important role in tumorigenesis and metastasis (51). For example, tumor suppressor miR-195-5p inhibits GATA3-mediated secretion of IL-4 in CRC cells and ultimately M2-like tumor-associated macrophages polarization by suppressing the NOTCH2 expression (52). Zhao et al. verified that exosomal miR-934-induced M2 macrophage polarization promotes CRC liver metastasis through activation of the CXCL13/CXCR5 axis in CRC cells (53). Similarly, Wang et al. found that exosomal miRNAs (miR-25-3p, miR-130b-3p, miR-425-5p) promoted CXCL12/CXCR4-induced CRC liver metastasis by enhancing M2 polarization of macrophages (54). Conversely, TAMs promote CRC cells proliferation and invasion by secreting transforming growth factor-β (TGF-β) that inhibits miR-34a expression and upregulates VEGF (55). Based on these, miRNAs that interact with immune cells also provide a new perspective for us to understand the progress of CRC.

#### 3.1.2 Cancer Associated Fibroblasts

Cancer associated fibroblasts (CAFs) are also one important component of TME, which is usually an important part of the tumor matrix (56). A large amount of evidence shows that many miRNAs are related to CAFs in CRC (57). CAFs can affect the TME of CRC by secreting a variety of chemokines and cytokines, to promote the metastasis and diffusion of cancer cells (57). Yang et al. confirmed that miR-31 can regulate the proliferation, metastasis and radiosensitivity of CRC cells by inhibiting autophagy of CAFs (58). In addition, HIF-1α up-regulates miR-210 affects the expression of VMP1, thereby improving the migration and invasion of CAFs. Metastasis of cancer associated fibroblasts from the CRC primary promotes tumor metastasis (59).

It is worth noting that CAFs can also mediate the expression level of miRNA by transferring extracellular vesicles directly to CRC cells (60, 61). Zheng et al. found that CAFs-derived extracellular vesicles can transfer SLC4A4-targeted miR-224-5p to CRC cells and promote the proliferation, migration, invasion and anti-apoptosis of CRC cells (62). Exosomes are another form of extracellular vesicles (63). Hu et al. found that the exosomes secreted by CAFs led to the increased expression of miR-92a-3p in CRC through the Wnt/β-Catenin signaling pathway to promote cell stem, EMT and metastasis in CRC (64). These suggest that CAFs are an important part of the TME that promotes the development of CRC.

#### 3.1.3 Mesenchymal Stem Cells

Mesenchymal stem cells (MSCs) are one of the main components of TME and can promote tumor angiogenesis and metastasis (65, 66). MSC-derived extracellular vesicles may play a role in the progression of CRC. For example, Li et al. have shown that MSC-exosomal miR-3940-5p inhibits invasion and EMT of CRC cells as well as growth and metastasis of tumors through targeting ITGA6 and TGF-β1 inactivation (67). Zhao et al. found that MSC-derived extracellular vesicles transmitting miR-34a-5p suppress tumorigenesis of CRC through c-MYC/DNMT3a/PTEN axis (68). Zhang et al. found that inhibiting miR-424 in bone marrow MSC-derived exosomes suppresses tumor growth in CRC by up-regulating TGFBR3 (69).

#### 3.1.4 Endothelial Cells

When the nutrition and oxygen provided by existing blood vessels cannot meet the needs of tumor growth and invasion, tumors will produce angiogenesis-related factors to change the local microenvironment and form new blood vessels (70). Endothelial cells (ECs) are the basic components of blood vessels (71). Previous data suggest that certain miRNA are key determinants of endothelial cells behavior during angiogenesis (72, 73).

Vascular endothelial growth factor A (VEGFA), which controls the proliferation, survival and migration of endothelial cells, is the main regulatory factor for the germination of new blood vessels (71). miR-125a-5p inhibits the ability of human umbilical vein endothelial cell (HUVEC) tube formation through VEGFA/VEGFR2 signaling pathway, and inhibits CRC progression, which has been further verified by mouse model (74). Moreover, miR-150-5p in CRC can inhibit HUVEC tube-forming ability and inhibit CRC tumor progression by targeting VEGFA in CRC through VEGFA/VEGFR2/Akt/mTOR signaling pathway (75).

Exosomal miR-25-3p regulates endothelial cells by targeting KLF2 and KLF4 to promote vascular permeability and angiogenesis, which can promote CRC metastasis (76). In addition, cancer cells-derived exosomal miR-27b-3p can be transported to vascular endothelial cells, where targeted p120 and vascular endothelial cadherin (VE-Cad) destroy the integrity of vascular endothelial cell junction. It also increases vascular permeability and result in promoting circulating tumor cell-mediated CRC metastasis (77).

#### 3.1.5 Cytokines

Differential expression of cytokines, chemokines and ncRNAs can regulate TME in the process of tumor progression (78). Pro-inflammatory cytokines play significant roles in CRC-related cachexia, including tumor necrosis factor α (TNFα), which is involved in the maintenance and homeostasis of immune system, inflammation and host defense (79). The overexpression of miR-19a can enhance the ability of TNF-α to induce spindle-like morphological characteristics and significantly upregulation the expression of N-cadherin, Fibronectin and Vimentin induced by TNF-α, and induce EMT to promote the invasion of CRC cells (80).

The interleukin-17 (IL-17) family is a Th17 cell-derived pro-inflammatory cytokine family involved in numerous human diseases (81). IL-17C has been shown to promote tumor progression by increasing epithelial cell survival and tumor angiogenesis in CRC (82, 83). Lee et al. demonstrated that IL-17C produced VEGF *via* the STAT3/miR-23a-3p/SEMA6D axis can cause intestinal endothelial cells to promote angiogenesis in the tumor environment (84).

### 3.2 LncRNAs in TME

**Figure 3:**
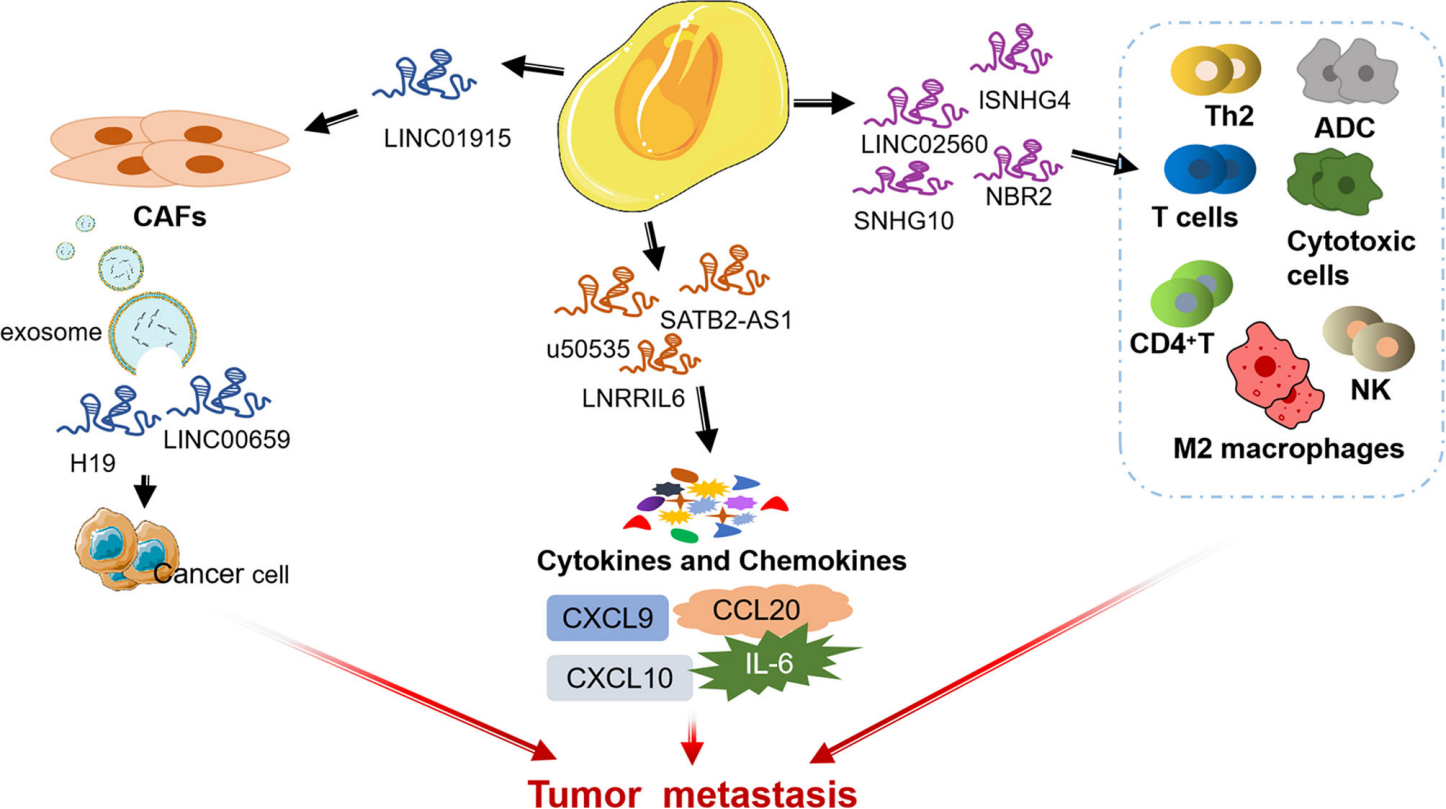
lncRNAs involved in the physical regulation of CRC in TME.

LncRNA is a kind of RNA with a length of more than 200 nt and no protein coding ability (85, 86). Recent studies have shown that lncRNAs play a vital role in CRC by affecting TME (87, 88). Next, we summarize some lncRNAs that influence CRC progression through regulating the different components of TME (**Figure 3**).

#### 3.2.1 mmune Cells

There is increasing evidence that lncRNAs are indispensable in immune responses by regulating immune cells (89–92). LINC02560 expression in CRC was significantly and negatively correlated with the infiltration of four immune cells (Th2 cells, T cells, ADCs, and Cytotoxic cells). Tumor with high LINC02560 expression reduced cellular immunity and antigen-presenting capacity in the microenvironment and participated in the progression of CRC (93). Ning et al. found that lncRNA SNHG4 induces CACO2 cell-CD4+ T cell interaction by targeting miR-144-3p and induces CD4+T cell apoptosis through the PD-1/PD-L1 immune checkpoint (94). In addition, exosomal lncRNA SNHG10 derived from CRC cells inhibits the activity and cytotoxicity of NK cells and promotes the immune escape of CRC cells by up-regulating the expression of INHBC (95).

LncRNAs mediate the regulation of TME by TAMs to affect tumor progression (96, 97). One study showed CRC cell-derived exosomes transport RPPH1 to macrophages, mediate the M2 polarization of macrophages and affect TME, thereby promoting the metastasis of CRC cells (98). In addition, Lai et al. found that up-regulation of lncRNA NBR2 in macrophages could inhibit M2 macrophages polarization and thus prevent the proliferation and metastasis of CRC cells (99).

#### 3.2.2 Cancer Associated Fibroblasts

LncRNA can affect crosstalk between malignant cells and CAFs during tumorigenesis (100, 101). LINC00659 metastasized from CAFs to cancer cells through exosomes and promoted CRC cell proliferation, invasion, migration, and EMT progression through the miR-342-3p/ANXA2 axis (102). Zhou et al. revealed that LINC01915 can inhibit the conversion of normal fibroblasts (NFs) into CAFs *via* the miR-92a-3p/KLF4/CH25H axis, and inhibit angiogenesis, thus preventing tumor growth (103). Moreover, CAFs contribute to promoting the stemness and chemoresistance of CRC by transferring exosomal H19 act as a competitive endogenous RNA sponge for miR-141 to activate the β-catenin pathway in CRC (104). In a separate study, CAFs can induce the up-regulation of lncRNA UCA1 and activate the proto-oncogene mTOR, thereby significantly stimulating the proliferation and migration of CRC cells (105).

#### 3.2.3 Cytokines and Chemokines

Interleukin-6 (IL-6) in TME is a pleiotropic cytokine, which plays an important role in the regulation of the immune system (106). As an important mediator of inflammatory reaction and activator of STAT3, IL-6 can reduce the apoptosis of cancer cells (107). LNRRIL6 can be used as an IL-6 activator to activate STAT3 by up-regulating IL-6 in the microenvironment, protecting CRC cells and promoting their proliferation (108).

LncRNA can regulate CRC tumor metastasis and affect tumor immune microenvironment by targeting some chemokines in TME of CRC (such as CC chemokine subfamily and CXC chemokine subfamily) (109, 110). For example, lncRNA SATB2-AS1 inhibits the expression of TH1 chemokines CXCL9 and CXCL10 affects the tumor immune cell microenvironment in CRC by regulating SATB2 (111). In addition, others found that CCL20 secreted by tumor cells is one of the main chemokines in TME, and lncRNA u50535 can regulate CCL20 expression and affect CCL20/CCR6/ERK signaling, leading to CRC tumorigenesis (112, 113).

In summary, lncRNA mediates the process of CRC by acting on cytokines in TME, and new insights on the function of cytokines in CRC help to promote the effective application of cytokine regulatory therapy in CRC.

### 3.3 CircRNAs in TME

**Figure 4:**
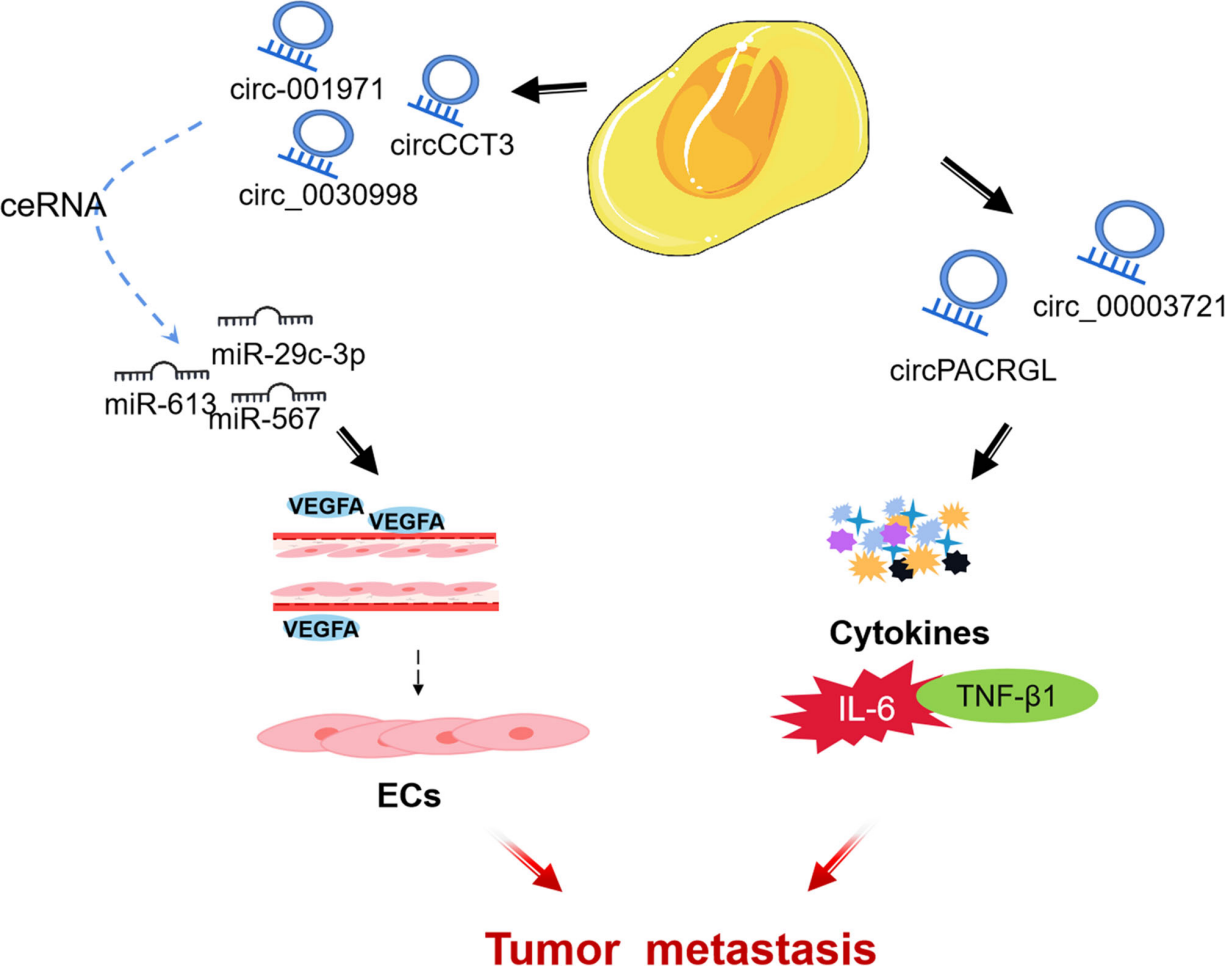
circRNAs regulates TME of CRC.

CircRNA is a subgroup of endogenous ncRNA (114). Some circRNAs are rich in microRNA (miRNA) response elements and can act as competing endogenous RNAs, binding miRNAs and thereby inhibiting miRNA functions and regulating gene expression (115, 116). With the advent of high-throughput sequencing, many circular RNAs have been successfully identified and have been confirmed to be involved in many biological regulations in the TME of CRC (117). Nonetheless, the biological functions of circRNAs in CRC are largely still intricate and unclear (118). Accordingly, understanding the mechanism of circRNAs in TME of CRC may be used as a diagnostic and prognostic biomarker, as well as a potential therapeutic target for CRC. Next, we review some circRNAs that influence CRC progression through regulating the different components of TME (**Figure 4**).

#### 3.3.1 Endothelial Cell

CircRNA can be used as an important process for ceRNA to affect the angiogenesis of ECs and mediate the growth and metastasis of CRC tumors by regulating miRNA. Chen et al. found that circ-001971 acts as a ceRNA to attenuate miR-29c-3p induced VEGFA inhibition, and through TME affects HUVEC tube formation, thereby exacerbating the proliferation, invasion, and angiogenesis of CRC (119). Similarly, Li et al. found that circCCT3 is highly expressed in human clinical CRC tumors, and regulates VEGFA expression and enhances the metastasis of CRC by sponging miR-613 (120). In addition, located in the cytoplasm of CRC cells, circ_0030998 was found to regulate VEGFA in CRC by sponging miR-567, promoting HUVECs formation and CRC cells proliferation (121).

#### 3.3.2 Cytokines

TGF-β1 (transforming growth factor -β1), a subfamily of TGF-β, inhibits N1 neutrophil in TME but promotes N2 neutrophil differentiation and cancer development (122). Exosomal circPACRGL promotes CRC cell proliferation, migration, and invasion, as well as N1-N2 neutrophil differentiation progression of CRC *via* the miR-142-3p/miR-506-3p-TGF-β1 axis (123). In addition, Liu et al. found that circ_0000372 up-regulated the expression of IL6 through spongiform miR-495, and activated the STAT3 pathway to promote the proliferation, migration and invasion of CRC (124).

## 4 Perspective: ncRNAs as Potential Targets for Colorectal Cancer

To date, the role of ncRNAs in radiotherapy and chemotherapy suggests that they can be used as new therapeutic targets for CRC (125–128). Over the past few decades, the treatment of CRC patients has changed significantly. In addition to traditional radiotherapy and chemotherapy, therapeutic targeting of TME has become a promising cancer treatment due to the critical role of TME in regulating tumor progression (4, 129). The dynamically complex TME provides favorable conditions for tumor growth, and tumor evolution and resistance to anticancer therapy are mediated through dynamic interaction with TME (130–133). The study of ncRNAs and TME also provides reliable evidence for their potential application in the clinical treatment of CRC.

A large number of studies have confirmed that ncRNAs can have multiple biological functions in regulating the onset and progression of cancer by affecting components or being affected in the TME (32, 114, 134–138). Regulatory T cells (Tregs) are known for their immunosuppressive effects, and targeting Tregs are effective method to increase chemical sensitivity (139–141). Studies have shown that miR-208b secreted by CRC cells is sufficiently delivered to receptor T cells to promote Treg amplification, tumor growth and oxaliplatin resistance by targeting programmed cell death factor 4 (PDCD4) (142). miRNA-124-3p inhibits the expression of PD-L1 by regulating STAT3 signaling pathway, and then promotes the anti-cancer response of CRC cells mediated by Tregs to inhibit tumorigenesis (143). Crosstalk between CAFs and cancer cells in the TME, making CAFs potentially important targets for stroma-based therapy in CRC treatment (4, 144). For example, Hu et al. confirmed that CAF-derived exosomal miR-92a-3p could be directly transferred to CRC cells to promote its metastasis and resistance to 5-FU/L-OHP (64). Moreover, exosomal miR-24-3p can be transferred from CAFs to colon cancer cells and down-regulate CDX2/HEPH axis, resulting in significantly enhanced resistance of colon cancer cells to methotrexate (MTX) (145). Deng et al. found that lncRNA CCAL transferred from CAFs to cancer cells through exosomes promoted the resistance of CRC cells to oxaliplatin (146). Yang et al. creatively found that the circEIF3K/miR-214/PD-L1 axis mediated the progression of CRC induced by hypoxia through CAF, providing a theoretical basis for the development of new targeted therapies for CRC (147). Furthermore, Zhou et al. found that LncRNA MIR155HG induced polarization of CRC cell M2 macrophages by regulating ANXA2, promoted CRC progression, and enhanced CRC cell resistance to oxaliplatin (148). Above evidences suggest that ncRNAs can play a role in the development of drug resistance in CRC, and the promotion of drug resistance can significantly reduce the efficacy of drugs and increase the failure rate of treatment with anticancer drugs such as oxaliplatin and 5FU (149, 150). This indicates that various ncRNAs in TME can be potential therapeutic targets to overcome drug resistance.

Actually, exosomes are widespread in all body fluids and can be detected and used as markers for the early CRC diagnosis (151). In addition, exosome ncRNAs are protected by lipid bilayer encapsulation and can be prevented from being degraded by ribonucleases in blood, which has a great possibility of being used as a carrier of therapeutic drugs (152, 153). Relevant exosome engineering technologies have been used to treat tumors by delivering tumor-inhibiting exosome ncRNAs (154–156). For example, modified exosome is used for co-delivery of 5-FU and miR-21 inhibitors (miR-21i) targeting CRC cells, which effectively reverses the drug resistance of tumor cells and remarkably enhances the toxicity of 5-FU-resistant cancer cells (157). These suggest exosomes can be used as carriers of therapeutic drugs and may guide changes in clinical practice.

In summary, above evidences suggest that ncRNAs play an important role in CRC therapy by modulating TME to enhance potential antitumor therapeutic targets. The function and potential clinical application value of ncRNAs in TME are worthy of affirmation. Their research will provide new ideas for targeted tumor therapy in the future and may be further explored in future research.

## 5 Conclusion

In conclusion, this review comprehensively discussed the roles of ncRNAs such as miRNAs, lncRNAs, circRNAs in TME of CRC and involved multiple biological processes. In this study, we affirmed that the aberrant expression of ncRNAs in the TME can directly or indirectly promote the proliferation, migration and drug resistance of CRC tumor cells. Although research on TME in ncRNAs and CRC is very limited to date, it offers broad prospects for cancer diagnostic and therapeutic applications. Further exploring the great potential and unresolved problems of ncRNAs and TME as potential therapeutic targets in clinical applications, we believe that more CRC patients will benefit in the future.

## Data Availability Statement

The original contributions presented in the study are included in the article/supplementary material. Further inquiries can be directed to the corresponding author.

## Author Contributions

ZW and QJ were involved in the conception of the study. ZW were involved in writing the article. QJ critically revised the manuscript. All authors have read and approved the final manuscript.

## Funding

This study was supported by the Clinical Medicine + X Project of Affiliated Hospital of Qingdao University (grant no. 66 to QJ).

## Conflict of Interest

The authors declare that the research was conducted in the absence of any commercial or financial relationships that could be construed as a potential conflict of interest.

## Publisher’s Note

All claims expressed in this article are solely those of the authors and do not necessarily represent those of their affiliated organizations, or those of the publisher, the editors and the reviewers. Any product that may be evaluated in this article, or claim that may be made by its manufacturer, is not guaranteed or endorsed by the publisher.
